# Supplementary material for: Are carbon emissions trading and green financial instruments synergistic? -Comprehensive quantitative research based on content analysis
Source: PLoS One. 2024 Mar 7;19(3):e0298601. doi: 10.1371/journal.pone.0298601 (PMC10919601; doi:10.1371/journal.pone.0298601)
Supplement: S1 Data — (ZIP) [file pone.0298601.s001.zip › Data/地级市Z_F工作报告.zip!/╨┬╜«/╬┌┬│─╛╞δ╩╨/└┤╫╘╣1⁄2╓┌║┼í╛┬φ┐╦╩2╛▌═°í┐_2014.docx]

各位代表：

　　现在，我代表市人民政府，向大会报告工作，请予审议，并请各位政协委员、列席代表提出意见。

　　一、2013年工作回顾

　　过去的一年，在自治区党委、自治区人民政府和市委的坚强领导下，在市人大、市政协的监督支持下，我们团结带领全市各族干部群众，全面贯彻落实党的十八大、十八届三中全会、自治区党委八届四次、六次全委（扩大）会议和市委十届五次全委（扩大）会议精神，坚持“稳中求进、进中求变、变中求好、好中求快”的经济发展总基调，积极应对复杂多变的宏观经济环境和下行压力，攻坚克难、变革创新、敢于担当、真抓实干，圆满完成了市十五届人民代表大会第二次会议确定的目标任务，为首府跨越式发展和长治久安奠定了坚实基础。

　　（一）主要经济指标实现“三年翻一番”

　　预计实现地区生产总值2400亿元，增长15%；全社会固定资产投资1280亿元，增长26.7%；社会消费品零售总额970亿元，增长16.3%；全口径外贸进出口总额120亿美元，增长15%；地方财政收入400.7亿元，增长26.1%；财政性总支出518.3亿元，增长23.6%；全社会用电量290亿度，增长16%；居民消费价格指数103.6%，实现了涨幅控制在4%以内的目标；城镇居民人均可支配收入20780元，增长13%；农牧民人均纯收入12065元，增长16.5%。主要经济指标在2012年“三年翻一番”的基础上，2013年又实现“三年翻一番”。尤其是人均地区生产总值突破1万美元，达11346美元，实现了历史性跨越。

　　新型工业化快速推进。工业增加值794亿元，增长14.7%，其中先进装备制造业、战略性新兴产业和高新技术产业实现工业增加值占全市30%以上。经济技术开发区（头屯河区）成为全疆首家工业总产值千亿园区，中小企业经济增速达30%。实施重点工业项目53个，上海大众5万辆轿车、新特能源1.2万吨多晶硅等项目建成投产，伊泰煤基多联产、神华煤基新材料等项目相继开工建设。新建工程技术研究中心14个、重点实验室7个、科技孵化器6家，培育创新型企业23家。连续荣获全国科技进步先进城市称号，被列为全国智慧城市建设示范试点。高新技术产业开发区（新市区）成为全疆首个国家知识产权试点园区。国家及自治区名牌产品总数达85个，驰（著）名商标达136件。创建国家及自治区循环经济试点园区3家，淘汰落后产能项目12项，规模以上工业企业万元工业增加值能耗下降12%。

　　服务业加快发展。积极推进国家服务业综合改革试点，完成服务业、物流业和城市商业网点规划修编，高端商务、总部经济加快发展，服务贸易扎实推进，会展、高铁、白鸟湖等新区一批城市综合体项目开工建设。启动会展片区金融城建设。乌拉泊国际物流基地农副产品物流配送中心建成启用。推动服务业提档升级，国家级钻级酒店达55家，特色餐饮街（区）达8条，特色餐饮进社区示范点达500个，城市连锁便利店达1042个，“放心肉”品牌连锁店（点）达361个，获批全国城市共同配送试点城市。主办承办中国清真美食文化节、冰雪风情节、文博会等大型展会76个。天山大峡谷成功创建国家5A级景区。全年接待国内外游客1950万人次，增长11%；旅游总收入270亿元，增长11.5%。

　　农业农村工作扎实推进。新增蔬菜生产面积1.1万亩，高效节水农业4万亩，新建温室大棚8000亩，创建国家级畜禽、水产养殖示范场（区）8个。启动百万肉羊（牛）生产体系建设，各类牲畜出栏155万头（只），增长19.1%。国家级农业科技园区获批启动。市级农业产业化龙头企业达26家，农民专业合作社达478家。农产品质量安全检（监）测点达323个。全面实施农村安全饮水及“两居”工程建设，建成安居富民房5470套，新（续）建农村公路112公里，解决农村5900人安全饮水问题。

　　（二）社会民生改善成效显著

　　全面完成改善民生15个方面120件实事。市财政用于民生方面的支出达261.7亿元，增长35.6%，占公共财政预算支出的74.1%。完成933个无物业管理老旧小区楼体、管网及附属设施综合改造，惠及30多万各族群众。改造提升主次干道77条，整治背街小巷250条，新（改）建公厕726座，智能垃圾房200座。建成覆盖中心城区的停车诱导系统和2座立体停车库，设置分时段免费停车路段38条。开工建设5个社会福利中心和4个日间照料中心，新（改）建120个社区老年活动中心，发放高龄津贴、政府购买居家养老服务补贴等2365.5万元。加大食品药品监督力度，开展重点行业及重点民生商品的价格监管和专项整治工作。加大市级肉菜储备力度，严格实施限价销售措施并适时投放，社区蔬菜直销点达500个，牛羊肉直销点达100个，居民消费价格指数涨幅低于全疆平均水平。

　　就业和社会保障工作扎实推进。新增城镇人员就业8.2万人，城镇登记失业率3.4%。开展各类职业技能培训9.7万人次，农村富余劳动力转移就业7697人次，“零就业”家庭保持24小时动态清零。发放小额担保贷款2.8亿元，实现创业带动就业1.5万人。实施专项人才计划和人才工程，引进各类高端紧缺人才1089人。积极推动社会保障“一卡通”建设，发放社会保障卡59万张，社会保险参保614万人次。企业退休人员养老金连续9年提高，社会化管理服务率达98%。城镇职工最低工资标准达1320元。城乡低保标准提高至每月360元和180元，发放低保金8354万元，各类专项救助金6142万元。

　　居民住房保障体系进一步完善。实施棚户区改造项目27个，新建房屋10224套、153万平方米。加大保障性住房建设力度，新开工建设公共租赁住房4005套、廉租房5000套、经济适用房2522套。6232户住房困难家庭通过公开摇号取得了保障房资格，外来务工人员首次纳入保障范围。完成公共建筑抗震加固工程7.55万平方米。

　　教育、卫生、体育等各项社会事业加快发展。稳步实施优质教育资源北扩工程，市第一中学、第十三中学新校区开工建设。全面推进教育强区（县）创建工作，5个区基本完成义务教育学校标准化建设任务，2个区分别通过国家和自治区义务教育均衡发展评估验收。新（改）建学校6所。新增公办幼儿园9所，认定普惠性幼儿园82所。落实“两免一补”等各类补助资金1.06亿元。完成基层医疗机构布点规划，完善医疗卫生服务网络，乡镇卫生院达27所，村卫生室190个；社区卫生服务中心达59所，社区卫生服务站223个。疾病预防控制体系进一步完善，基本药物制度覆盖所有基层医疗机构。人均基本公共卫生服务经费补助和新农合人均筹资标准不断提高，重大疾病医疗保障范围持续扩大。人口和计划生育各项政策全面落实，人口出生率为8.77‰。广泛开展全民健身活动，成功举办乌鲁木齐市第三届运动会，市第五小学卫冕全国青少年校园足球赛冠军。新闻出版、防灾减灾、外事、工商、质监、气象、地震、人防、地方志、档案工作取得新进展。老龄、妇女儿童、残疾人和红十字事业都取得新成绩。

　　（三）现代化国际城市建设步伐加快

　　城乡规划不断完善。编制完成城北新区核心区、南山旅游基地等控制性详规和老城区改造提升整体规划，重点商圈改造和物流企业外迁稳步实施，新区路网等基础设施建设加快推进。全面完成农村规划编制工作，实现了全市所有乡镇、村的规划全覆盖。

　　城市基础设施建设超常规推进。投入79.9亿元实施了24项重点道路交通项目，会展大道二期、喀什路东延、南湖路北延等一批项目建成通车。“田”字路二期工程顺利竣工，全立体交通骨架构建完成，道路通行能力提升30%。完成地铁1号线勘察设计和东绕城高速公路、高铁新客站前期征收工作，三屯碑快速公交枢纽项目加快推进。城市公共交通不断完善，新增公交线路297.7公里，更新公交车辆979辆，完成“田”字路快客2条线路14对站点建设，大容量快速公交系统建设项目获得中国人居环境范例奖。第十三届全国冬运会冰上运动中心建设顺利推进。大西沟水库建成蓄水，城北新区供水工程实现向城区供水，实施了供排水管网改建三期、南郊净水厂、西山水厂扩建等项目。完成餐厨垃圾资源化利用和无害化处理一期等项目，实施建筑垃圾再生利用、生活垃圾焚烧发电项目，垃圾无害化处理率达到91.4%。城市管理网格化、精细化水平不断提高，环卫保洁、冰雪清除工作达到国内领先水平。

　　大气污染治理成效显著。投入137亿元，新（改）建大型锅炉房80座，安装燃气锅炉250台5500蒸吨，新增燃气供热能力5000万平方米，中心城区实现清洁能源供热全覆盖。建成南部集中供热及配套燃气管线55公里。米东区大气污染治理工作全面推进。实施既有建筑供热计量及节能改造项目3661.6万平方米。完成苇湖梁电厂、神新煤矸石电厂及3家企业燃煤火电机组关停工作，取缔关停高污染企业360家，完成20家企业限期治理任务。严格机动车环保标识管理，全面执行“黄标车”限行和“无标车”禁行。提升建筑工地及道路清扫扬尘控制水平，彻底关停42家堆煤场。全年减排二氧化硫3.7万吨、烟尘1.1万吨、氮氧化物4.8万吨，减少燃煤消耗700万吨，空气质量优良率达83%以上，空气质量由全国74个重点监控城市排名末位上升到前20位左右。

　　以园林绿化为重点的生态建设迈上新台阶。全年新增绿化面积6.2万亩、大苗基地2万亩、封育抚育3万亩，新建小游园、小绿地156块，小水面20个。突出绿化景观建设，建成天山公园，着力打造“绿谷”、华光街等一批绿化精品工程，绿化景观水平和文化内涵进一步提高。乌板乌水公路两侧生态防护林示范建设通过国家和自治区验收。全市建成区绿化覆盖率达到36.16%，创建国家园林城市顺利通过验收。全面启动创建国家卫生城市工作，深入开展环境综合整治，实施6条精品示范路和77条城市主干道综合整治，完成道路修补及改造提升87.75万平方米。清洗粉刷楼体281栋，规范户外广告6662块，拆除违法建筑121.42万平方米，亮化各类建筑85座。水源地保护工作全面加强，全面实施柴窝堡湖生态恢复工程，完成乌拉泊水源地10项综合环境整治工程。砂场关停治理强力推进，关停砂场2家，治理恢复乌拉泊砂场130公顷。水资源保护和节水力度不断加大，通过国家节水型社会试点城市验收。

　　（四）改革开放取得新突破

　　改革创新深入推进。简政放权取得重大突破，取消、下放171项行政审批事项，压缩108项行政审批事项办理时限，审批事项和办理时限均压缩50%以上；坚持责权利相统一，下放市级管理权限379项，以区为主的建管体制不断完善。融资平台建设及融资功能进一步完善，市级融资主体达到15家，多元化融资格局逐步形成，区（县）融资能力不断增强。完成市商业银行增资扩股任务。非公有制经济和中小微企业健康发展，制定促进民间投资工作方案，鼓励民间资本进入市政公用和社会事业领域；加强中小微企业服务平台建设，创新开展各类融资业务，为中小微企业提供贷款108亿元。完成种牛场及长胜大队整体接收工作，拓展了城市发展空间。启动事业单位分类改革，完成事业单位类别划分认定工作。认真落实天山北坡经济带发展规划，兵地融合和乌昌经济一体化加快推进。积极开展乌鲁木齐发展战略等重大问题专项研究。

　　对外开放步入新阶段。新增国家和自治区级外贸出口基地6个，进出口超亿美元企业达20家。实施招商引资项目321项，引进法国液化空气集团、大连万达等一批国际国内大企业、大集团。引进区外到位资金517亿元，增长39%；实际利用外资2.23亿美元，增长15.6%。第三届中国—亚欧博览会经济技术合作项目及贸易合同签约总额达1506.11亿元，较上届增长31.31%。成功举办丝绸之路经济带城市合作发展论坛，8个国家24个城市参会并签署《乌鲁木齐共识》，合作项目协议签约总额8.6亿美元。

　　（五）文化建设扎实推进

　　围绕构建社会主义核心价值体系，深化民族团结进步工作和“热爱伟大祖国、建设美好家园”主题教育活动，举办“马兰精神”、“喀喇昆仑精神”等巡回报告会16场，开展各类群众性宣讲活动108场。文明城市创建工作深入推进，大力开展公民道德建设、机关文化建设和现代都市文明教育，开展服务行业和窗口单位服务达标竞赛活动，各行业文明水平进一步提升，顺利通过自治区文明城市测评。推进基层科普行动计划，促进全民科学素质不断提高。文化惠民工程深入推进，“六馆”建设步伐加快，新增社区文化室30个，全面完成农家书屋和“直播卫星户户通”建设任务，免费放映公益电影8963场，开展文化惠民演出300余场次，举办各类群众性文化活动1300余场次。文化创意产业园开工建设，“7坊街”创意产业集聚区等文化品牌影响力不断扩大。

　　（六）社会大局持续稳定

　　面对依然严峻复杂的稳定形势，我们坚定不移地落实自治区党委和市委各项维稳部署，确保了首府社会政治大局持续稳定，实现了“两个坚决”和“两个确保”目标。“三项重点工作”不断加强，始终保持对“三股势力”严打高压态势，发现、打击、防范和处置“四种能力”不断提升。依法加强宗教事务管理，坚决查处“三非”活动，坚决抵制宗教极端思想渗透。完善公共信息发布制度，及时澄清突发事（案）件真相，消除群众疑虑。不断创新流动人口和出租房屋管理模式，推行流动人口管理限时工作法、“一证通”制度和房屋租赁证试点工作。“大政法委”和三级应急指挥体系高效运转，突发事件现场处置“六快”办法在全疆推广。“三个基础”进一步巩固，新建18个派出所，新设6个街道办事处和121个社区，新建社区全部达到“十有”条件。重点社区“数字化”管理平台全部建成，社区“四化”建设日臻完善，步巡、车巡、网巡相结合的防控体系初步形成。强化安全生产责任制落实，大力推进安全生产标准化、隐患排查治理体系和监管能力建设，深入开展“打非治违”等专项整治工作，各类安全生产事故下降15.8%。健全矛盾调解和信访工作机制，信访总量下降10%。深入开展“军民共建美好精神家园”和以“五送五进”为主的双拥共建活动，军政军民团结进一步巩固。

　　（七）政府自身建设全面加强

　　不断深化依法行政，深入推进“六五”普法工作。制定实施《乌鲁木齐市人民政府工作规则》，提请市人大常委会审议地方性法规草案3件，制定政府规章4件。自觉接受人大法律监督和政协民主监督，积极听取各民主党派、工商联、无党派人士和人民团体意见。办理人大代表议案9项16件、建议156件，政协委员提案413件，办复率100%。加强政务公开和网络问政，受理市长专线2.68万件，办结率99.41%；市长信箱884件，办结率99.15%；网络问政3850项，回复率100%。启动区、市两级行政服务中心（公共资源交易中心）项目建设。行政监察、审计监督进一步强化，预防和惩治腐败工作成效明显，加强对关键环节的制约监督，以“零容忍”态度严查腐败案件。认真贯彻落实中央“八项规定”和自治区党委、市委“十项规定”，深入开展机关干部赴基层转变作风、服务群众活动，各类会议减少50%，文件减少8.2%，“三公”经费支出下降13.52%。不断加强机关效能建设，全面落实绩效考评工作，深入开展民主评议政风行风和创建示范窗口单位活动，政府公信力、执行力和服务群众的能力进一步增强。

　　各位代表，回顾2013年的工作，我们深深感到：所有这一切，是自治区党委、自治区人民政府和市委坚强领导的结果，是全市各族人民共同努力和各方面大力帮助支持的结果。在此，我代表市人民政府，向全市各族干部群众，向驻乌部队、武警官兵、公安民警、兵团职工，向人大代表、政协委员、各民主党派人士，向所有关心支持首府改革、发展与稳定的各界朋友，表示衷心的感谢并致以崇高的敬意！

　　在总结成绩的同时，我们也清醒地看到前进道路上面临的困难和问题：一是外部环境复杂多变，保持经济快速增长压力较大，增长的质量和效益需要进一步提高。二是市场经济体系还不完善，改革开放亟待全面深化。三是综合交通枢纽建设任务艰巨，“五城同创”时间紧迫，生态环境治理和保护力度需要不断加大。四是社会治理体系还不完善，基本公共服务能力还不强，群众在就业、出行、物价、物业等方面仍有不少意见。五是反恐维稳形势依然严峻，维稳常态化、长效化机制需要进一步巩固。六是政府职能转变还不到位，服务群众的能力和水平仍需进一步提高。对此，我们将在今后的工作中采取措施切实加以解决。

　　二、2014年主要工作

　　2014年是我们全面落实党的十八届三中全会精神、全面深化改革的第一年，也是顺应各族群众新期待、在全疆率先全面建成小康社会的攻坚之年。

　　全市工作的总体要求是：全面贯彻落实中央、自治区党委和市委一系列重要安排部署，紧紧围绕长治久安抓发展，抓住并用好历史机遇，坚持稳中求进、改革创新，着力推动经济转型跨越发展，提高经济增长质量和效益，着力优化城市环境，加快现代化国际城市建设，着力加强以现代文化为引领，繁荣城市特色文化，着力防治大气污染和保护生态环境，提高生态文明建设水平，确保民生不断改善，确保物价基本稳定，确保社会持续稳定，确保实现发展目标，努力推动科学跨越、后发赶超，努力打造全疆引领之地、首善之城。

　　经济社会发展的主要目标是：地区生产总值增长13%；工业增加值增长12%；全社会固定资产投资增长27%；社会消费品零售总额增长14%；全口径进出口贸易总额增长10%；公共财政预算收入增长16%；城镇居民人均可支配收入增长13%；农牧民人均纯收入增长16%；居民消费价格总水平涨幅控制在4%以内；新增城镇就业7.8万人，城镇登记失业率控制在3.7%以内。实现上述总体要求和主要目标，我们要重点做好以下工作：

　　（一）围绕实现长治久安，确保社会大局持续稳定

　　坚决贯彻中央、自治区党委和市委一系列重大决策部署，不断强化“五个观点”和“五个宁可”思想，进一步增强忧患意识，树立底线思维，坚持综合施策、标本兼治、强化治本，切实提高社会治理和维护稳定能力。

　　一是进一步强化维稳常态化措施。毫不松懈地抓好“三项重点工作”，打牢“三个基础”，发挥三级应急处置指挥体系和“大政法委”作用，始终保持严打高压态势。加强流动人口管理，制定流动人口和出租房屋管理条例实施细则，推广社区“限时工作法”。强化社区“四化”管理，坚持车巡、步巡、网巡相结合，提升基层维稳防控管理能力。加强社会治安综合治理，严密防范和依法惩治各类违法犯罪活动。不断深入查找维稳工作中的漏洞和薄弱环节，并及时加强和改进，确保实现“两个坚决防止”目标。

　　二是依法加强宗教事务管理。严格落实“三管两制一负责”制度，加强街道社区民族宗教工作队伍建设，提高宗教场所自我管理能力。充分发挥爱国宗教人士作用，教育引导信教群众自觉在法规制度规范下进行宗教活动。坚决贯彻落实自治区党委11号文件和市委63号文件精神，以“去宗教极端化”为重点，依法查处非法宗教活动、非法宣传品和网上非法煽动，强化网络舆论引导和管控，坚决遏制宗教极端思想渗透。

　　三是深入推进民族团结进步。牢牢把握各民族共同团结奋斗、共同繁荣发展的主题，深入开展民族团结进步创建和主题教育实践活动，大力宣传民族团结先进典型，促进各民族互相尊重、互相学习、互相帮助，不断巩固和发展平等、团结、互助、和谐的社会主义新型民族关系。特别是广泛开展形式多样、健康向上、入脑入心的民族团结教育活动，有针对性地加强青少年、流动人口等重点人群宣传教育。

　　四是不断创新社会治理体系。加快健全社会组织和公众参与的社会治理体系，发挥好政府和社会两方面治理作用。健全重大决策社会稳定风险评估制度，发挥人民调解、行政调解、司法调解联动作用，加强信访工作，有效预防和化解社会矛盾。完善安全生产责任体系，严格落实安全生产准入制度，全面推进隐患排查治理体系、安全生产标准化和监管能力建设，切实提升重点行业领域安全生产保障和事故防范能力，着力减少各类安全事故的发生。积极支持国防和军队现代化建设，做好国防动员、人民防空和民兵预备役工作。加强国防教育，深入开展双拥共建活动，争创全国双拥模范城“八连冠”。

　　（二）推动全面深化改革取得突破

　　贯彻落实市委十届七次全委（扩大）会议确定的首府全面深化改革总体目标：按照市场主导、充满活力、民主法治、公平正义、开放包容、团结和谐的要求，进一步解放思想、解放和发展社会生产力、解放和增强社会活力，全面推进治理体系和治理能力现代化，在重要领域和关键环节改革上取得决定性成果，形成与城市现代化、国际化相适应的系统完备、科学规范、运行有效的制度体系，争当新疆全面深化改革排头兵，努力打造丝绸之路经济带“五大中心”。

　　当前和今后一个时期，要着力在十个方面重点改革上取得突破。一是坚持和完善基本经济制度，在发展混合所有制经济上取得突破。健全国有资产监管体制；逐步推动国有企业“三个转变”；大力发展非公有制经济，清理废除各种不合理规定，推动非公企业参与城市基础设施建设和运营管理；制定相关扶持政策，推进股权投资类企业健康发展；大力发展混合所有制经济，鼓励国有资本、集体资本、非公有资本等交叉持股。二是坚持和完善现代市场体系，在政府职能转变上取得突破。加大简政放权力度；强化发展规划、政策、标准等制定和实施；完成新一轮市、区（县）两级政府机构改革任务；加快事业单位分类改革；清理规范市场准入限制，建设统一开放的市场体系；废除部门行业妨碍公平竞争的各类制度性规定，打破部门行业垄断；整合规范各类要素市场，加快公共资源交易中心建设；深化财税体制改革，建立财权与事权相匹配的财政体制，完善市、区（县）土地征收、储备和收益分配机制；推进诚信首府建设。三是加快构建开放型经济体系，在建设丝绸之路经济带“五大中心”上取得突破。促进与中亚城市“五通”，启动“五大中心”规划建设；争取批准设立亚欧经贸合作试验区；加强与国内外地区和城市合作，共同建设产业合作园区；尽快建立综合保税区，推进通关便利化；协调争取国际航班“经停”等政策，协调航空、铁路、公路等部门拓展国际陆航联运线路；推进区域性国际金融中心建设，抓紧建设结算中心、外汇交易中心、股权交易中心和大宗特色资源产品期货市场，制定实施扶持金融产业发展政策。四是深化农业农村改革，在构建现代都市农业体系上取得突破。按照城市规划，慎重稳妥积极推动农业农村各项改革工作；积极引导农村人口市民化；构建现代农业产业体系，支持各类专业合作社和专业大户，支持农业龙头企业发展，培育职业农民；推进农村金融和保险服务。五是健全和完善城市管理体制，在构建城市治理体系上取得突破。继续下放权力，把各区建成完全责任政府；构建“市级统筹、区县落实、协调配合、运行高效”的城市治理新体系；健全城市综合管理标准评价体系和监管考核体系。六是深化社会领域改革，在推进基本公共服务均等化上取得突破。健全促进就业创业机制；健全社会保障和救助体系；深化教育综合改革和医药卫生体制改革。七是加快推进文化体制机制创新，在增强文化创新发展活力上取得突破。加快文化管理体制改革；健全互联网管理领导体制；培育合格文化市场主体；扩大政府公共文化采购。八是深化行政执法和司法体制改革，在促进社会公平正义上取得突破。规范行政执法；完善行政执法程序；建立执法司法业绩档案；健全司法权力运行机制、司法救助和社区矫正机制，完善法律援助制度。九是加快生态文明制度建设，在环境保护和生态修复上取得突破。健全大气污染长效治理和区域联防联控机制；编制城市生态环境总体规划和功能区规划；健全资源性产品价格形成机制；建立区（县）生态环境差别化考核机制。十是健全权力运行制约和监督体系，在坚持用制度管权管事管人上取得突破。推进办事公开制度化、标准化建设；完善干部绩效考核和选拔任用监督制度；探索大部门监察机构建设；建立改进作风常态化制度。

　　（三）推动经济提质增效升级

　　统筹转方式、调结构、促增长、抓改革，加快打造“六大产业基地”，全力提升经济增长的质量和效益，全面激发经济增长内生动力。

　　一是提升工业经济发展水平。围绕先进制造业基地、高新技术产业基地和战略性新兴产业基地建设，加快完善园区配套设施，培育壮大优势产业，促进产业向高端方向发展。支持高新技术产业开发区（新市区）创建国家创新型特色园区，经济技术开发区（头屯河区）创建国家服务外包示范基地，全力打造全疆新型工业化引领区。强化12个产业集群规划引导，促进风电、汽车、光伏、电子新材料及现代煤化工等产业延伸产业链。继续推进钢铁冶金、石油化工、纺织等传统支柱产业改造升级，促进骨干龙头企业提质增效。协调推进三一西北重工2600台工程机械、伊泰540万吨煤基多联产、莱沃科技5万吨多功能宽幅农用膜等30个重大项目建设。积极推进国际纺织品服装商贸中心、“云计算”产业基地、新疆软件园等重点示范项目。

　　二是促进服务业优化升级。扎实推进国家现代服务业综合试点城市和共同配送试点城市建设，创新服务业发展体制机制，优化服务业空间布局。加快现代服务业基地建设，大力推进企业总部、金融保险、会展物流、文化旅游等现代服务业，加快金融城及大型城市综合体建设。高起点规划、高水平建设城南经贸合作区，将其打造成为城市生态新区和开放合作的高地。加快推进五大物流园建设，科学布局一批现代物流项目。全力做好第四届中国—亚欧博览会的服务保障和组团参展工作，全年举办展会达到90个以上。着力扩大消费需求，拓展消费领域，推动消费结构升级。加快改造提升传统商贸服务业，进一步创新商业模式，鼓励发展电子商务、连锁经营等新兴商贸业态，加快形成便利化、多样化的居民生活服务网络。推进南山旅游基地规划建设，全面整合乌鲁木齐县与达坂城区旅游资源，抓好特色旅游项目开发建设，加快向国内一流旅游景区目标迈进。加大旅游宣传推介力度，开拓丝绸之路沿线旅游合作，力争实现接待国内外游客增长10%。

　　三是加快推进农业农村工作。完善乡镇农村整体规划，加快水西沟、板房沟、达坂城等中心镇建设。全面落实市委加快推进农业农村工作实施方案，投资20亿元用于农村产业增收、“两居”工程、社会事业、基础设施和基层组织建设，促进城乡一体化。加快构建现代农业产业体系，积极发展设施农业、节水农业和休闲观光农业，蔬菜生产面积达到26万亩，农业高效节水面积达到10万亩，新（改）建设施农业6000亩。实施百万肉羊（牛）生产体系建设，新（改）建标准化肉牛羊养殖小区（场）50个。推进土地承包经营权流转。全力推进“两居”工程，建设“两居”住房2.8万套。加快推进米东区老龙河治理等工程，分批实施农村道路、供排水、垃圾污水处理、电力、燃气等项目建设。建立改善村容村貌、完善农村基础设施建设管理长效机制。积极吸引各类优秀人才到农村工作。

　　四是全方位扩大对外开放。深化与丝绸之路经济带沿线城市全方面经济合作，加快同周边区域基础设施互联互通建设，办好第九届丝绸之路国际城市市长论坛。建立完善跨区域城市发展协调机制，认真落实天山北坡经济带发展规划，加强“乌昌石”城市群经济协作互动，争取尽快启动“五大经济圈”城际铁路规划建设，推动区域经济一体化发展。支持兵团第十二师“三化”建设，促进兵地融合发展。创新招商引资模式，着力引进一批国内外500强企业和产业链配套企业，确保实际利用外资和引进区外到位资金增长15%以上。大力发展出口导向型经济，积极发展服务贸易和服务外包产业。进一步扩大机电产品、高新技术产品、地产品出口规模，加快建设外贸公共信息服务和电子商务平台、国际口岸和出口加工基地平台。支持企业“走出去”开拓国际市场，鼓励和引导企业在中亚国家兴办实体，建立售后服务机构和营销网点，占领向西开放的制高点，不断提高国际化城市的经济外向度。

　　五是坚持创新驱动发展。实施创新驱动、知识产权和名牌带动战略，推进国家创新型试点城市和知识产权示范城市建设，加快国家级农业科技园区建设。健全科技投入持续增长机制，加大对产业共性技术、应用型技术研究开发的支持力度，加速科技成果转化应用，充分发挥科技引领支撑经济社会发展作用。围绕提升产业核心竞争力，实施重大科技专项，支持建立企业主导的产业技术创新战略联盟，引导创新要素向企业集聚。加快创新平台建设，推进10家重点科技孵化器建设，新建工程技术研究中心和重点实验室5个，新增高新技术企业、创新型企业20家。争创驰（著）名商标11件，名牌产品10个。不断完善信用担保等服务体系，促进融资性担保公司、小额贷款公司健康发展，扶持中小微企业向专业化、高科技化和集群化方向快速发展。

　　（四）全力保障和改善民生

　　一是千方百计做好就业工作。落实积极的就业政策，发挥企业吸纳就业主渠道作用，实现大中专毕业生就业率达到85%以上。深化国家创业型城市建设，全面落实鼓励自主创业的扶持政策，形成政府激励创业、社会支持创业、劳动者勇于创业的新机制，促进创业带动就业。加快市级人力资源市场和50个基层公共就业服务所（站）建设，创建充分就业社区40个。强化就业技能、岗位技能和创业能力培训，完成各类职业培训9.2万人次，创业培训1.1万人次。转移农村富余劳动力6000人次。大力实施人才强市战略，深化产教融合、校企合作，完善选拔培养机制，壮大高素质、高技能人才队伍，加快构筑首府人才高地。

　　二是不断提高社会保障水平。加快完善统筹城乡的社会保障体系建设，推进社会保险关系转移接续，不断扩大社会保险覆盖面。建立正常的养老金待遇调整机制，推进城乡居民大病医疗保险试点工作。完善城乡低保标准正常调整机制和分档差额补助政策，推进社会救助体系建设，提高困难群众生活水平。加快养老福利园区建设，完善老年人基本生活津贴和养老服务补贴制度，积极构建以居家为基础、社区为依托、机构为支撑的养老服务体系。加快发展社会福利事业和慈善事业。

　　三是加大保障性住房建设和棚户区改造力度。健全保障性住房分配和使用监管机制，新建各类保障性住房12000套，其中廉租房5000套、公租房4500套、经济适用房2500套。实施棚户区改造6000户、60万平方米，抓好回迁安置、市政配套建设和小区物业管理服务工作。加强防灾减灾体系建设，提升地震、地质、气象等灾害防御能力，继续实施生命线工程及公共建筑抗震加固工程。

　　四是做好稳定物价和便民服务工作。完善社区蔬菜副食品直销点运营管理机制，丰富肉菜、粮油等副食品供应，扩大市级肉菜储备和投放，保持居民生活必需品价格基本稳定。理顺食品药品监管体制，加大市场监管力度，切实保障食品药品安全。加快“公交都市”建设，加大公交运力投入，调整优化公交线路，新建大容量快速公交系统7号线，启动“田”字路快客运营。建设新一代智能交通指挥管理系统，疏通道路微循环系统。加大停车场开发建设力度，加强车辆违规停放整治工作。巩固933个无物业小区综合改造成效，制定物业管理条例实施细则，规范物业服务市场，实行物业企业分类评定制度，建立物业服务价格调整机制，完善房屋维修基金管理办法，强化街道社区对辖区物业企业监管。探索建立无物业小区管理基金，采取资金补贴等方式引导物业公司介入，切实解决无物业小区管理服务问题。

　　五是大力发展教育、医疗卫生、体育等社会事业。全力实施教育强区（县）战略，加快中小学校标准化建设，运动场标准化率达到80%以上，全市80%的区（县）完成义务教育标准化建设任务，40%的区（县）完成义务教育均衡发展任务。扩大优质教育资源覆盖面，着力解决中心城区学校“大班额”问题，逐步缩小区域、城乡、校际差距。大力发展学前教育，加快发展普惠性民办幼儿园。扩大职业教育规模，培养更多实用型技能人才。稳步推进双语教育。严格落实教育用地，城市新区和新建小区开发必须按规划保证教育用地。做好校园周边环境整治和校车管理工作，切实保障校园安全。加强教师队伍建设，提高师德师能水平，努力办好人民满意的教育。加强公共卫生和医疗服务体系建设，推进会展、高铁片区医院建设，切实提升27所乡镇卫生院、190个村卫生室和59所社区卫生服务中心、223个社区卫生服务站的服务能力。强化传染病和地方病的预防控制，完成健康教育七大任务。加强流动人口计生服务和管理，提高出生人口素质。落实全民健身计划，广泛开展群众性体育活动，加强社区公共体育设施工程和农牧民体育健身工程建设。积极做好自治区第十三届运动会、第八届少数民族传统体育运动会组织参赛工作。促进妇女儿童、残疾人和红十字事业，推动统计、外事、工商、质监、人防、地方志、档案等各项社会事业蓬勃发展。

　　（五）高水平做好城市规划建设管理工作

　　一是不断完善城乡规划布局。围绕打造现代化国际城市，进一步优化城市空间布局，提升城市功能品质。积极做好轨道交通沿线、城北新区、城南经贸合作区等控制性详规的编制，完成城市总体规划成果的报批。坚持依规划建设新区、改造老区，进一步完善七大片区发展规划，推进会展、高铁、白鸟湖、古牧地、城北新区、城南经贸合作区和甘泉堡经济技术开发区建设，完善路网和基础设施，优化公共资源配置，协调服务万达、宝能、绿地、红星美凯龙等大型城市综合体项目落地建设，增强新区承载力和吸引力。全面强化规划管控和政策引导，实施老城区改造提升工程，切实缓解老城区人口、交通和社会管理压力。集中在老城区打造一批特色历史文化街区。完善规划和土地市级长效管理机制，强化批后管理，有效遏制违法建设行为。

　　二是强力推进重大基础设施建设。投资300亿元，实施160项基础设施建设项目。围绕构建区域综合交通枢纽，协调做好航空、铁路和高速公路建设，加快推进高铁新客站建设，全力确保年内高铁正式通车。重点实施地铁1号线、克南高架东延、西山路高架二期、新医路西延、城北主干道延伸等项目，加快做好东二环、国道216线仓房沟段项目征收工作。完成三屯碑快速公交枢纽项目建设。实施中心城区供水瓶颈改造、排水管网改建及观园路区域供水工程等项目。协调启动水磨沟、鲤鱼山等220千伏输变电工程，建成凤凰—西山—东郊750千伏输变电工程。加快推进第十三届全国冬季运动会冰上运动中心建设，完善周边基础设施配套建设，确保年内竣工。

　　三是进一步提高城市管理水平。按照国家卫生城市创建标准，扩大环境综合整治范围，重点抓好环境卫生、景观美化和社会秩序整治等工作，解决硬件设施不足和城市管理薄弱问题。广泛动员驻乌单位和各族市民参与创建活动，形成共建美好家园的强大合力，确保各项指标达到国家卫生城市标准。实施以“田”字路沿线建筑景观整治为重点的主要街道景观整治。推广垃圾分类及无害化处理，全面做好市政设施维护和机械化清扫保洁工作，道路清扫和冰雪清除实现机械化全覆盖。新建智能化垃圾房408座，密闭式垃圾房100座，道路亮灯率达到95%以上。新建粪便无害化处置项目，公厕实现全部免费开放。加快智慧城市建设，完成基础地理信息、人口信息、机构信息三大公共基础数据库建设。健全城市公共安全应急保障体系，提高安全运行保障能力，提供安全、稳定、优质的公共产品和服务。

　　（六）推动文化大发展大繁荣

　　一是加强社会主义核心价值体系建设。围绕创建全国文明城市，坚持以现代文化为引领，广泛开展社会主义核心价值体系和中华民族伟大复兴中国梦的宣传教育，大力弘扬新疆精神，引导各族干部群众坚定理想信念、凝聚改革共识，共同团结奋斗。深入推进公民道德建设和机关文化建设，持续开展精神文明创建活动，抓好文明服务、文明秩序、文明环境建设，全面提高公民素质和社会文明程度。以政府部门和窗口服务行业为重点，推动服务礼仪、服务标准与国际接轨。加强城市外语标识系统建设，推进社会各领域服务便利化和国际化。

　　二是大力发展文化事业和文化产业。加快建设完善的现代公共文化服务体系，推进“六馆”等重大文化设施项目建设，实施政府购买演出、公益电影放映、广播电视“村村通”等一系列文化惠民工程。繁荣文化艺术精品创作，大力开展群众性文化活动，丰富各族群众文化生活。加快编制文化产业发展规划，实施重大文化产业项目带动战略，推进文化产业基地和特色文化产业园区建设。充分挖掘丝路文化、民俗文化、玉石文化等特色文化，促进文化与科技、教育、旅游深度融合。加快培育骨干文化企业、创建自主文化品牌，积极发展文化创意、数字出版、动漫游戏等新兴产业。进一步加强文化遗产保护，加大文化市场监管力度。

　　三是扩大文化交流。秉承各民族优秀传统文化，积极发展首府特色文化，实施政府文化交流项目，广泛开展对外文化交流与合作，加强对外传播能力建设，提升首府文化的竞争力和影响力。

　　（七）加快生态文明建设

　　一是强力推进大气污染治理。进一步巩固大气污染治理成果，切实提高市民健康水平。完善大气污染联防联控体系，严格落实“八个一律”措施，实施16个治理改造项目，全面完成污染物总量控制目标，全年空气质量达标天数保持在300天以上。加快大气污染重点区域专项治理，全面推进米东区大气污染治理和乌鲁木齐县燃煤锅炉天然气改造项目，力争实现全市清洁能源全覆盖。加快工业企业污染治理工作，推进电厂关停治理和重点企业污染防治设施建设，强制推行企业清洁生产。对各类工地、清扫保洁及散装物料储运实施最严格的防尘措施，坚决治理扬尘污染。加大机动车尾气治理力度，严格落实“黄标车”限行和“无标车”禁行措施，年内淘汰1.4万辆“黄标车”。实施供热系统节能改造试点项目建设，完成既有居住建筑供热计量及节能改造360万平方米，改造更新老旧供热管网280公里。进一步提高新建建筑节能设计标准，大力发展绿色建筑，积极推进住宅产业化。

　　二是提升城市生态绿化水平。紧紧围绕建设美丽首府，巩固国家园林城市创建成果，新增绿化面积3.5万亩，人工造林1.5万亩，封育抚育2万亩。启动环城生态圈建设，大力推进三北防护林、退耕还林等林业生态工程建设。加大道路景观绿化力度，高标准完善城市绿网体系，继续打造一批绿化景观精品。新建小游园、小绿地100个，新增一批小步道、小水面。抓好水源地、湿地及城市周边生态修复，大力推进柴窝堡湖、一号冰川生态涵养与保护，建设头屯河、乌鲁木齐河、水磨沟河、白杨河四条河流生态廊道。完成大西沟水库管道引水工程，尽快实现“三库联调”，保障城市供水安全。加快实施一批污水处理及再生水利用工程，力争再生水回用率达到40%。启动国家生态园林城市创建工作，推进经济技术开发区（头屯河区）国家生态示范园区建设。

　　三是推进节能减排和资源节约利用。围绕建设低碳城市，狠抓节能减排和循环经济发展，加快淘汰落后工艺和过剩产能，推进清洁生产和绿色低碳发展，积极申报国家节能减排综合示范城市。推进大浦沟循环经济产业园建设，继续实施生活垃圾焚烧发电、餐厨垃圾资源化利用和无害化处理、建筑垃圾再生利用等工程。全面贯彻实施最严格的耕地保护、水资源管理和环境保护制度，推进节约集约利用资源。

　　（八）进一步加强政府自身建设

　　新形势、新任务对政府自身建设提出了更高的要求，我们要切实转变政府职能，深化行政体制改革，创新行政管理方式，依法履行政府职能，努力建设职能科学、结构优化、廉洁高效、人民满意的服务型政府。

　　一是强化服务意识，践行群众路线。深入开展党的群众路线教育实践活动，切实解决政治坚强和“四风”方面存在的突出问题，以优良的作风树立政府形象，密切同群众的联系。按照权责一致、分工合理、决策科学、执行顺畅、监督有力的要求，加快转变政府职能，加强市场监管和各类公共服务提供。继续深化行政审批制度改革，最大限度减少政府对微观事务的管理，全面清理现有各项行政审批、许可事项，限期完成清理和减少任务。加快区、市两级行政服务中心（公共资源交易中心）建设步伐，努力打造一流的投资发展环境，把首府建成“审批事项最少、效率最高、服务最优”的城市之一。大力推进绩效考评工作。加强政务公开和政务服务，推进决策公开、管理公开、服务公开、结果公开，充分保障群众的知情权、参与权、表达权和监督权。

　　二是强化法治意识，坚持依法行政。坚持用制度管权管事管人，让人民监督权力，让权力在阳光下运行。加快构建决策科学、执行坚决、监督有力的权力运行体系，严格按照法定权限和程序行使权力。主动接受人大法律监督和政协民主监督，认真听取各民主党派、工商联、无党派人士和人民团体的意见，自觉接受社会舆论监督。健全决策机制和程序，完善规范性文件、重大决策合法性审查机制，提高政府决策科学化、民主化、法制化水平。规范行政执法程序和执法自由裁量权，做到严格规范公正文明执法。深入推进“六五”普法、法律援助、社区矫正和人民调解工作，努力建设更加规范有序、高效便民、公开透明的法治政府。

　　三是强化廉政意识，坚持从严治政。健全惩治和预防腐败体系，完善行政权力运行制约和监督机制，落实廉政建设责任制。加强效能监察和行政问责，强化重点领域、重点部门和重点项目的监察审计，坚决纠正损害群众利益的不正之风，严肃查处各类违纪违法案件。深入落实中央“八项规定”和自治区党委、市委“十项规定”，健全改进作风常态化制度，保持艰苦奋斗，厉行勤俭节约，着力改进会风文风，严格控制“三公”经费支出和楼堂馆所建设。加强公务员职业道德、履职能力和行政执行力建设，发扬实事求是、实干兴邦的作风，坚定理想信念、严守政治纪律、把住廉政底线、不谋私利特权，切实做到干部清正、政府清廉、政治清明。

　　四是强化实干精神，狠抓工作落实。全面落实市委决策部署，实现经济社会发展目标，狠抓五个落实。第一，围绕长治久安抓落实。紧紧抓住难得的历史机遇，坚定发展信心，精心谋划发展的措施和办法，努力实现经济有质量、有效益、有速度、可持续地增长。第二，改革创新抓落实。坚持变化变革、敢于担当，以逢山开路、踏石留印、抓铁有痕的精神狠抓落实，打好全面深化改革攻坚战。第三，突出重点抓落实。把主要精力用在解决重点问题、推动重要工作上来，统筹协调，通盘谋划，切实做到突出重点，把握关键，落实到位。第四，求真务实抓落实。大力弘扬求真务实、埋头苦干作风，坚持“只有努力才能改变，只要努力就能改变”，着力推动市委确定的各项决策部署落到实处，把追求实效贯穿于所有工作全过程，确保各项既定目标任务圆满实现。第五，优化作风抓落实。牢固树立“民生优先、群众第一、基层重要”的理念，切实转变工作作风，密切联系群众，把人民群众的期待作为工作的动力，全心全意为群众办实事、办好事，真正把中央、自治区和我市的各项惠民政策落到实处，让改革发展成果惠及各族群众。

　　各位代表，立足新起点，应对新挑战，推进新发展，实现新跨越，这是时代赋予我们的历史重任和光荣使命。让我们更加紧密地团结在以习近平同志为总书记的党中央周围，在自治区党委、自治区人民政府和市委的坚强领导下，奋力拼搏，真抓实干，为率先全面建成小康社会，实现跨越式发展和长治久安而努力奋斗！
